# Pemphigoïde bulleuse paranéoplasique associée à un cancer bronchique

**DOI:** 10.11604/pamj.2015.21.248.6034

**Published:** 2015-08-06

**Authors:** Fatima Safini, Nezha Tawfiq

**Affiliations:** 1 Service de Radiothérapie-Oncologie, Centre Hospitalier Ibn Rochd, Casablanca 1, Casablanca, Maroc

**Keywords:** Syndrome paranéoplasique, pemphygoide bulleuse, cancer, paraneoplastic syndrome, bullous pemphigoid, cancer

## Image en medicine

**Figure 1:**
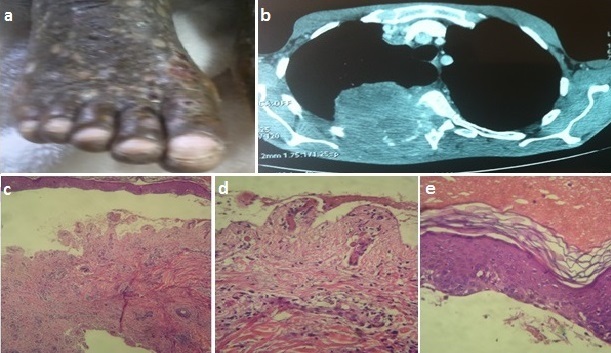
A) lésions bulleuses en phase de regression; B) aspect d'image infiltrant le parenchyme pulmonaire lobaire inférieur droit; C) décollement jonctionnel dermo-épidermique; D) plancher de la bulle dermique; E) toit épidermique normal

La pemphigoïde bulleuse paranéoplasique est une entité peu reconnue par rapport au pemphigus paranéoplasique. Son association à une maladie cancéreuse fait toujours l'objet de controverse. Nous rapportons l'observation d'un patient de 46 ans qui avait consulté pour des douleurs hémi-thoraciques droites évoluant dans un contexte d'amaigrissement chiffré à 10 kg en 6 mois. L'examen clinique retrouvait des lésions cutanées bulleuses très prurigineuses diffuses à tout le corps, sans atteinte ni du visage ni de la muqueuse buccale (Figure 1a). Le scanner thoracique avait révélé un processus tumoral du lobe inférieur du poumon droit (Figure 1b). Le diagnostic d'un cancer bronchique indifférencié à petites cellules a été porté sur une biopsie trans-pariétale. L’étude histologique réalisée sur des biopsies cutanées montraient un clivage de la jonction dermo-épidermique réalisant une bulle surmontée d'un épiderme sans nécrose ni acantholyse. Le derme papillaire sous et en périphérie de la bulle était le siège d'un infiltrat inflammatoire périvasculaire essentiellement lymphocytaire associé à des polynucléaires neutrophiles et éosinophiles. II existait en immunofluorescence directe des dépôts jonctionnels en IgG et C3. (Figure 1c, d, e). Le diagnostic retenu était celui de pemphigoïde bulleuse. Une chimiothérapie à base d’étoposide et cisplatine a été démarrée. Une régression des lésions cutanées de plus de 75% a été notée après deux cycles de chimiothérapie. A la lumière de ce cas et de la revue de la littérature, il nous paraît raisonnable lors de l'apparition d'une pemphigoïde bulleuse, de rechercher systématiquement un cancer sous-jacent par un examen clinique complet et des examens complémentaires orientés.

